# Protocol for a pseudo-randomized controlled trial to assess the impact of eco-driving assistance systems on bus drivers’ stress responses

**DOI:** 10.1265/ehpm.25-00259

**Published:** 2025-11-15

**Authors:** Maryline Krummenacher, Manosij Ghosh, Michelle C Turner, Irina Guseva Canu

**Affiliations:** 1 Department of Occupational and Environmental Health, Unisanté, University of Lausanne, Switzerland; 2 Environment & Health Unit, Department of Public Health and Primary Care, KU Leuven, Leuven, Belgium; 3 Barcelona Institute for Global Health (ISGlobal), Barcelona, Spain; 4 Universitat Pompeu Fabra (UPF), Barcelona, Spain; 5 CIBER Epidemiología y Salud Pública (CIBERESP), Madrid, Spain

**Keywords:** Eco-driving assistance technology, Public transport, Green transition, Occupational stress, Heart rate variability (HRV), Biological monitoring, Self-assessment

## Abstract

**Background:**

Technological innovations in the public transport sector are increasingly leveraged to support the goals of environmental sustainability and public health. Eco-driving assistance (EDA) systems represent one such intervention, aimed at reducing fuel consumption, emissions, and operating costs while improving passenger comfort. However, the potential unintended impacts of EDA technologies on driver health and well-being remain understudied. The EDA Trial, part of the EU-funded INTERCAMBIO project, seeks to evaluate whether the use of EDA systems may introduce new psychosocial stressors for professional drivers, with implications for occupational and public health.

**Methods:**

The EDA tested in this trial is called “NAVIG”. Buses will be assigned randomly. Operating EDA-equipped vehicle will be considered as intervention condition, operating vehicle without EDA as control. Each participant will be monitored for 10 working days maximum to accumulate at least 5 intervention shifts during the trial. Heart rate variability (HRV) will be continuously recorded during working hours to assess autonomous stress responses. The root mean square of successive differences (RMSSD) will be averaged over intervention and control shifts to enable within-subject comparisons between intervention and control conditions. Subjective stress levels will be evaluated using the self-report instruments: Cohen’s perceived stress scale at baseline and visual analogous scale at baseline and daily. Moreover, neuroendocrine stress biomarkers (salivary cortisol and cortisone) will be collected repeatedly across shifts, as additional outcomes. Mixed-effects models with participant’s ID as a random effect variable will be used to compare stress outcomes between EDA and non-EDA driving conditions. Models will be adjusted for potential confounders.

**Results:**

A sample size of 26–40 participants was estimated to provide 80% power (α = 0.05) to detect differences of 12–15% between conditions. Ethical approval was obtained from the Swissethics (CER-VD 2024-01573), and participant recruitment is ongoing, with 27 drivers enrolled as of June 2025.

**Conclusions:**

This study will provide empirical evidence on the potential health trade-offs associated with implementing eco-driving technologies in real-world settings. By assessing physiological and psychological stress responses to EDA, the trial supports a more integrated approach to environmental technology evaluation—one that considers not only energy efficiency but also the health and sustainability of the workforce.

**Trial registration:**

The trial was registered in the ClinicalTrials.gov database (NCT06688721)

## Background

Bus driver training and monitoring of their driving behaviors with respect to energy consumption, vehicle use and pollution as part of the so-called eco-driving programs has been foreseen as a possible solution to increasing public transport sustainability. Eco-driving is a series of driving techniques and maintenance procedures to achieve greater vehicle fuel efficiency [1]. Eco-driving training involves teaching techniques such as smooth acceleration, anticipatory braking, and optimal gear usage to reduce fuel consumption and emissions. Performance monitoring incorporates technologies designed to influence driver behavior in-cab, and monitoring techniques within the operator companies to promote continuous eco-driving. These technologies include vehicle telematics, which are complex systems integrating telecommunications and informatics to enable real-time monitoring of location, movements, status and behavior of a vehicle and/or driver. They also provide the user with up-to-the-minute knowledge of their fleet activities in one centralized, web-based interface [2]. Designed to reinforce the bus driver’s eco-driving behavior, these systems are called eco-driving assistance (EDA) [3].

Little is known on how the implementation of EDA systems in public transport may influence bus drivers’ well-being and working conditions.

Most studies on EDA implementation in public bus transport to date have focused on performance measurement regarding energy saving or CO_2_ emission but rarely have addressed EDA acceptance by drivers [3–10]. Importantly, no study has examined the EDA’s effects on bus drivers’ health and well-being.

In this study we aim to assess whether the use of EDA in public transport companies constitutes a new occupational stressor for bus drivers with ultimate harmful effects on their health and well-being. For this work we formulated the following research hypotheses. First, if EDA use acts as an additional stressor, driving with EDA would activate the body’s stress regulation systems, primarily of the autonomous nervous system (ANS) axis, whose effects on heart rate and HRV (usually a decrease in HRV) are immediate and can be measured within 1–5 seconds. If the trial showed that driving with EDA decreases the bus driver’s HRV in comparison to driving without EDA, it would indicate the inability of the ANS to adapt to the EDA presence in the bus drivers’ workplace as an effective stress regulation mechanism. Secondly, we hypothesize that driving with EDA would correspond to a higher psychological stress perceived by bus drivers at the end of the working day, compared to the working days of driving without EDA. Indeed, in addition to the continuous driving performance monitoring and feedback, the EDA of interest generates detailed reports informing bus drivers of their overall performance to ultimately improve their driving behavior. This self-training module may act as additional burden on the bus driver, especially in the case of unsatisfactory performance. Finally, we hypothesized that if EDA acts as a true stressor, it will also activate the Hypothalamic-Pituitary-Adrenal (HPA) axis of stress regulation via neuroendocrine pathway. Indeed, the HPA axis requires a more substantial or sustained stressor to be activated and takes minutes to hours because it involves hormonal signaling cascades. That would result in increased level of biomarkers (i.e., cortisol and cortisone).

## Methods

### Study design and setting

This is a pragmatic pseudo-randomized controlled trial using a within-subject study design [11]. This design enables a study participant to be exposed to the intervention (i.e., driving shifts with EDA) and serve as their own control (i.e., driving shifts without EDA), based on a randomly established sequence of intervention and control driving shifts.

The experimental phase of EDA Trial is set in a Swiss public bus transport company of the Fribourg canton (*Transports Publics Fribourgeois* - TPF) that has developed and exploits an EDA system. Given that the TPF has several bus centers, and that each bus driver is affiliated to one bus center, the trial will be conducted by center, one center at time for logistical and organizational reasons.

In all study steps, starting from design development, we insured the patient and population involvement (PPI) [12] by recruiting four sector managers. The sector manager is a particular status among senior bus drivers, acquired through professional promotion. Each sector manager is affiliated to two to four bus centers and is responsible for a group of bus drivers (between 70 and 185 bus drivers depending on the center). The sector manager is the first contact and reference for bus drivers for their daily work activities.

### Participant sampling, information and inclusion

To constitute a representative sample of the company bus drivers, we examined the employee demographics (sex and age distributions) based on the company human resources records and drafted the stratified sampling scheme. According to this scheme, the final sample should comprise 50 participants with at least 15% females and cover all age groups (See section *Sample size section*).

**Fig. 1:**
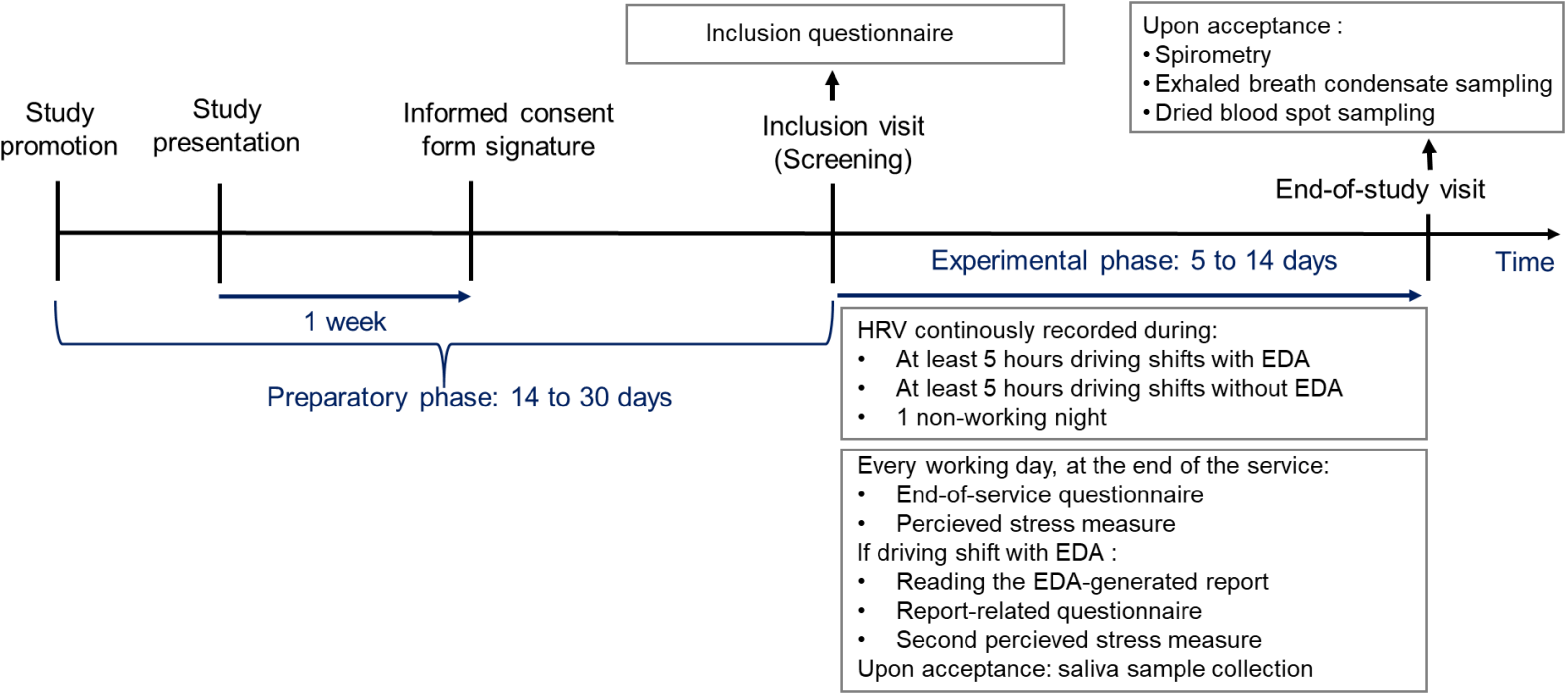
Study layout and timeline

To recruit the necessary number of participants, we co-designed the recruitment information leaflet and invitation message to be emailed by the internal company communication service. In parallel, the eligibility criteria and sampling scheme were presented to sector managers, who orally promote the study and motivate potentially eligible bus drivers within their sectors to attend at a study presentation session (Fig. 1).

Individual face-to-face detailed study presentation sessions are then organized at each bus center. During these sessions, the investigator explains the nature of the study, its purpose, the procedures involved, the expected study duration, the potential risks and benefits and any discomfort it may entail. At the end of the study presentation session, the research team provides a participant information sheet with the consent forms describing in detail the study. One week is given to the interested bus drivers to confirm their participation.

Each bus driver willing to participate in the study is invited to an individual inclusion visit. This visit is organized in alignment with the bus driver’s work schedule at the appropriate bus center. A dedicated study room is set up at each bus center, to host the individual inclusion visits and perform all study-related activities. At the beginning of the inclusion visit, the signed individual informed consent form is collected. Then, the research investigator screens the participant’s eligibility based on following criteria: 1-Being an active bus driver, 2-Having worked for at least 1 year at TPF, 3-Working at least 30 hours per week, 4-Not have planned work absences in the next 6 months that could interfere with the experiment organization (e.g., civil service, military service, long-term leave, retirement). The two exclusion criteria are: 1-Wearing a medical active implant (pacemaker, cardiac defibrillator, neurostimulator, other electronic implants) and 2-Known allergy or skin sensitivity to plasters.

Once the eligibility is confirmed, the investigator assigns each included participant a unique randomly generated ID code that will be used throughout the study to de-identify the collected data and thus ensure the participants’ privacy. This code is then entered into the study REDCap application, which allows the investigator to properly manage data collection procedures and participant follow-up.

REDCap generates a link to the electronic inclusion questionnaire that participant can complete using professional iPad or smartphone. The baseline questionnaire collects data on variables that might act as potential confounders, mediators or moderators in the exposure-effect relationship (See section *Covariates and potential confounders*).

After completing the baseline questionnaire, the participant receives the study material (i.e., measurement devices, tubes for saliva self-collection and the participant’s diary containing textual and visual information on study procedures). The investigator also provides detailed oral explanation and training on how to use them and answer participant’s questions. The measurement data recording is tested at the same time.

### Intervention to be tested

The EDA tested in this trial is called “NAVIG”. NAVIG has an added module on the bus dashboard displaying the driving quality scores as two varying bars, a green one for the ecological and energy consumption score and a blue bar for the passengers’ comfort score. NAVIG is connected to the onboard computer and has extra captors (accelerometers, gyroscopes, etc.) around the bus that measure several parameters. Based on the measured parameters, it continuously detects and records specific driving behaviors. Those behaviors and events take points out of the score, and the scores diminish visibly on the dashboard. After a certain time driving well (i.e., ecologically friendly), the scores increase again. In addition, the system generates a report, available on the NAVIG platform on bus driver’s professional iPad. This report provides time and GPS-related information on the driving quality during the driver’s trips. The report is generated in real time, but its delivery through the iPad can be delayed.

There are two types of indicators used by NAVIG. The first type of indicators are detected once a certain threshold has been surpassed, e.g. the system will detect a downhill acceleration above a certain threshold. The second type of indicators compare the bus driver’s driving behavior to the mean driving behavior on the same road segment, for instance the ratio of use time of brakes and retarder. In fact, using the engine brake allows for a smoother driving style and means less wear on the equipment. The indicators contribute to a different extent to the environmental and comfort score (weighting).

The use of NAVIG is currently voluntary, i.e., a bus driver may decide to switch it off, in which case there is no indicator display and no NAVIG reported generation. However, to test NAVIG and assess its potential effects on bus drivers’ well-being and health, participants will have to use NAVIG every time it is present in the allocated bus and to consult the generated report at the end of their work shift. Therefore, no intervention blinding will be possible.

### Primary outcome measurement

As a primary endpoint we will use heart rate variability (HRV) that represents the variation in time between adjacent heartbeats. HRV provides information on the autonomic nervous system and the balance between parasympathetic and sympathetic activity. An optimal HRV reflects the ability of the autonomic nervous system to adapt to body requirements and is advantageous, whereas a low HRV is indicative of fatigue, overtraining or health issues [13]. HRV represents an important health marker for stress researchers, popularity in work stress studies [14–16].

Clinical multi-lead electrocardiography is a traditional method of HRV measurement. Among wearable ECG devices measurement, Bittium Faros™ (Bittium Corporation, Oulu, Finland) is considered as a reference [17–19]. Participants will wear the Bittium Faros™ continuously during the working days throughout the study, using patch electrodes. Recording of HRV during at least 5 working days and reaching 5 hours of driving shifts with the intervention (i.e., bus with EDA) and 5 hours with the control (i.e., bus without EDA) is needed to obtain robust data. Moreover, participants will wear the Bittium Faros™ during one full non-working night to obtain their baseline values of HRV.

We will use the root mean square of successive differences (RMSSD) that reflects cardiac vagal tone [20, 21]. Contrary to other HRV indices, RMSSD is relatively free of respiratory influences [22].

### Secondary outcome measurement

As secondary outcomes we will measure perceived psychological stress and HPA axis hormones as biomarkers of stress. Both secondary outcomes will be assessed at the end of each working shift, that bus drivers call the end-of-service.

To measure perceived psychological stress, participants will use a visual analogous scale (VAS), graduated from 0 (no stress) through 100 (the highest perceived stress). This VAS has been successfully used in occupational settings and validated [23–25]. The VAS will be part of the electronic end-of-service questionnaire. Every working day during the experimental phase of the study, participants will receive a link to this questionnaire and answer questions characterizing the working day and encountered stressful events. Participant will also indicate whether they drove a bus with NAVIG at least one driving shift. If no, they will rate their perceived stress once, at the end of the questionnaire. If yes, they will rate their stress twice; before and after consulting the NAVIG report on their professional iPad. After the second stress rating, drivers will answer questions regarding the NAVIG report to indicate whether they agree or not with the NAVIG’s evaluation and scoring of their driving performance.

To measure stress biomarkers, saliva samples from bus drivers will be collected at the end of each working day, after filling in the end-of-service questionnaire. We will use pre-labelled Salivette^®^ swab self-collection device (Sarstedt, Nümbrecht, Germany) [26]. The participant will place the swab in the mouth where it easily absorbs saliva. The saturated swab is then placed back into the Salivette^®^ container, capped and stored in the freezer in the study room. Saliva samples will be stored at −80 °C until the shipment and salivary cortisol [27] and cortisone [28] concentrations will be measured using LC-MS/MS, considered as the reference method [29, 30].

### Covariates and potential confounders

HRV measurements can be influenced by several factors that have to be carefully monitored [31]. Individual factors such as age and biological sex and the influence of the circadian rhythm must be considered. We will have access to the work schedules of the bus drivers, allowing us to synchronize HRV analysis with the time of day and to inform the sickness absence. Moreover, we will collect data on traffic conditions, passenger numbers, and the bus model used in each shift to compare these parameters across the driving shifts of the same participants. Information on the bus model will allow us to control for differences in ergonomics and in exposure to physical and chemical hazards, using bus-ergonomics and bus-exposure matrices [32, 33].

Lifestyle factors such as alcohol consumption, physical activity, and smoking, can also impact HRV, and will be assessed through the inclusion questionnaire and the end-of-service questionnaire. Environmental factors such as temperature and solar ultraviolet radiation will be measured when possible using personal wearable devices: the iButton and SunSaver UV [34].

Finally, the presence of diagnosed diseases will be assessed using inclusion questionnaire, spirometry and biomarkers measured on the local pulmonary level in exhaled breath condensate (EBC) and on the systemic level in dried blood spot (DBS) samples. This will allow for assessing the early signs of diseases, which have not been yet diagnosed. Indeed, chronic obstructive pulmonary disease (COPD) is often prevalent among public transport workers [35, 36], although in a mildly symptomatic form [37]. Despite being an established method to monitor lung function and detect COPD [36], spirometry might be less sensitive than biomarkers measured directly in the lung lining fluid to detect COPD at early stages [37].

The EBC samples will be collected using the Turbo-Deccs^®^ (Medivac, Parma, Italy) and stored at −80 °C until analysis [38]. LC-MS/MS will be used for quantifying inflammation and oxidative stress biomarkers, as described in [39–42]. The DBS samples will be self-collected, using a pick-and-place on the fingertip to apply few capillary blood drops on 4 spots on a specific filter paper [43]. The DBS samples will be analyzed using V-PLEX Plus Proinflammatory Panel 1 Human Kit to measure biomarkers associated with the inflammatory response and immune system regulation, the cytokine-endothelial interaction pathway in vascular dysfunction.

### Experimental phase duration

Once the inclusion visit has taken place, the experimental phase of the study starts (Fig. 1). The total duration of the experimental phase depends on when the endpoints are reached but will last a minimum of 5 days and a maximum of 14 days. Participants will need to drive at least 5 hours with a bus with EDA and 5 hours with a bus without EDA. In addition, the recording on HRV during one non-working night is needed. Once the endpoints are reached or when the maximum duration of the study is reached, the study team will notify the participant that the study is over, and the end-of-study visit will be organized (Fig. 1).

### Sample size

The sample size estimation was focused on the primary outcome (log transformed RMSSD), assuming that a difference of about 12–15% between the two conditions (EDA vs. no EDA) is scientifically reasonable and clinically relevant [44–46]. The within person standard deviation (SD) of the differences of the log transformed RMSSD between a low-stress and high-stress situation was estimated at 0.64 ms [47]. To be able to determine a difference of 12% and 15% between the two conditions as significant with an alpha level of 0.05 and a power level of 0.80 in a paired sample t-test, the sample size needs to be between 26 and 40 participants, respectively. This corresponds approximately to a medium-sized effect (i.e., Cohen’s d of about 0.45–0.55). To be cautious and compensate for possible missing records, we plan to recruit a total sample of 50 participants.

### Statistical analyses

Within-person differences of the continuous log-transformed RMSSD for EDA vs. non-EDA driving shifts will be calculated and shown with SDs and 95% confidence intervals. The resting RMSSD average will be calculated based on the HRV measurement during one non-working night.

Mixed effects models with a random effect per participant (accounting for within subject correlations across repeated measures) and a random effect per day nested within the random effect per subject (to account for repeated measures per day within subjects) will compare RMSSD on EDA driving shifts versus on non-EDA driving shifts adjusted for the relevant subject-specific (e.g., age, sex, BMI), physiological (e.g., lung function parameters and biomarker level), exposure-related (e.g., solar UV, heat and cold), and driving shift specific (e.g., heavy traffic, rural vs. urban routes) covariates and potential confounders.

Potential bias that may arise from usage of different bus models. In case of different bus distributions, statistical models will be adjusted for exposures and bus characteristics which differ significantly. This adjustment will be possible by using the Swiss Bus Exposure Matrix [33] that contains exposure data on seven types of physical and chemical hazards and the Bus Ergonomics Matrix [32] that contains six scores of biomechanical and ergonomic constrains for 705 bus models that have been used in the last 40 years in Switzerland. All the statistical analyses will be performed using Stata and/or R software.

## Results

The trial started in February 2025. The experimental phase is ongoing and will continue until December 2025. In June 2025, 27 bus drivers were included. Most accepted providing saliva samples and performing optional tests.

## Discussion

### Study strengths and limitations

EDA Trial has a pragmatic design with the strength that the findings can be directly discussed to seek the necessary adjustments and their implementation in the partner company. The expertise in co-creation and shared decision developed within the EU INTERCAMBIO project [34] in addition to existent PPI activities planned in this trial will be valuable for seeking optimal solutions to preserve bus drivers’ health and well-being at work.

The fact that we evaluate only one kind of EDA in one single company can be seen as a limitation, since study results could not be generalized to all types of EDA. Indeed, each EDA has its own functioning and specific characteristics [48]. Moreover, the use of the EDA may also differ depending on how the company promotes it [5]. An EDA used on a voluntary base (e.g., like it is currently used in TPF) might not generate the same stress level as a mandatory use of EDA, especially if EDA’s rating results would influence the pay received by bus drivers and their future in the company [8, 10]. Conversely, supportive training, driver involvement in the rollout process, and a non-punitive feedback framework could ensure that the benefits outweigh the potential drawbacks [2, 3].

The fact that we rely on the self-collection of the HRV measurement data and biological samples could be seen both as an advantage and a limitation. Considering the irregularity of bus driving shifts and long working hours (70% of Swiss bus drivers have at least one working day of more than 10 hours a week and work during the weekend), the self-collection of data and samples offers higher flexibility, which might increase the participation rate by reducing the number of meetings with the research team and, consequently, the logistical and financial burden of the study. However, this could increase the between-subject and perhaps the within-subject variability if participants deviate from the standardized operational procedures (SOPs). To prevent this, we use the study presentation and inclusion visits for alerting and training participants to respect the SOPs. In addition, the study material (video-clip and participant journal) co-created within this study should help enforce adherence to the SOPs. As this issue has not been addressed in the literature yet, during the interview at the End-of-study visit we will explore whether the self-collection should be considered as a source of bias or an additional burden on the study participants.

As by Since our primary outcome is the heart rate variability, the collection of biological samples for biomarker measurement is optional. Given the planned sample size (50 bus drivers) and the likelihood that some participants will decline this option, the analysis of secondary and tertiary outcomes may lack statistical power, limiting the overall informativeness of the study. Furthermore, as bus drivers have irregular hours and night shifts, we decided against morning or repeated saliva sampling, despite the known circadian variation in cortisone and cortisol levels. Consequently, we acknowledge that the biomarker analysis is likely to serve an exploratory rather than an etiological purpose.

## Conclusion

EDA systems in passenger buses can help reduce air pollution by promoting smoother driving behaviors—such as gradual acceleration, optimized gear shifting, and reduced idling—which lower fuel consumption and associated emissions. For such systems to be effective, they must be user-friendly, integrated into bus drivers’ routines without increasing stress and workload, and supported by appropriate training, incentives, and organizational commitment to sustainable practices.

EDA Trial will gain insight to which extent the use of EDA NAVIG could align with this requirement. Monitoring HRV, neuroendocrine stress biomarkers and subjectively perceived stress will allow to gain the first experimental data and to measure the EDA’s effect on stress experienced by bus drivers in their working time. The focus on stress as a primary outcome of this trial, has an anticipatory preventive purpose, considering the pivotal role of stress in disease development and progression. Indeed, occupational exposure to stress significantly increases the risk of mental, respiratory, cancer, musculoskeletal, cardiovascular and metabolic diseases [49], which are highly prevalent among bus drivers [50–57]. Therefore, the introduction and widespread implementation of eco-driving technological solutions in public transport should only occur following a thorough evaluation of the associated risks and benefits for the health and well-being of professional drivers.

## Declarations

### Availability of data and material

There are no new data associated with this article.
